# Supplementary material for: Consistent Biomarkers and Related Pathogenesis Underlying Asthma Revealed by Systems Biology Approach
Source: Int J Mol Sci. 2019 Aug 19;20(16):4037. doi: 10.3390/ijms20164037 (PMC6720652; doi:10.3390/ijms20164037)
Supplement: Supplementary file 1 [file ijms-20-04037-s001.zip › ijms-566768-supplementary-XML/Supplementary Material S2-Reports of Quality control for ten datasets.zip!/Supplementary Material S2-Reports of Quality control for ten datasets/QC_report_for_GSE41861/hm.pdf]

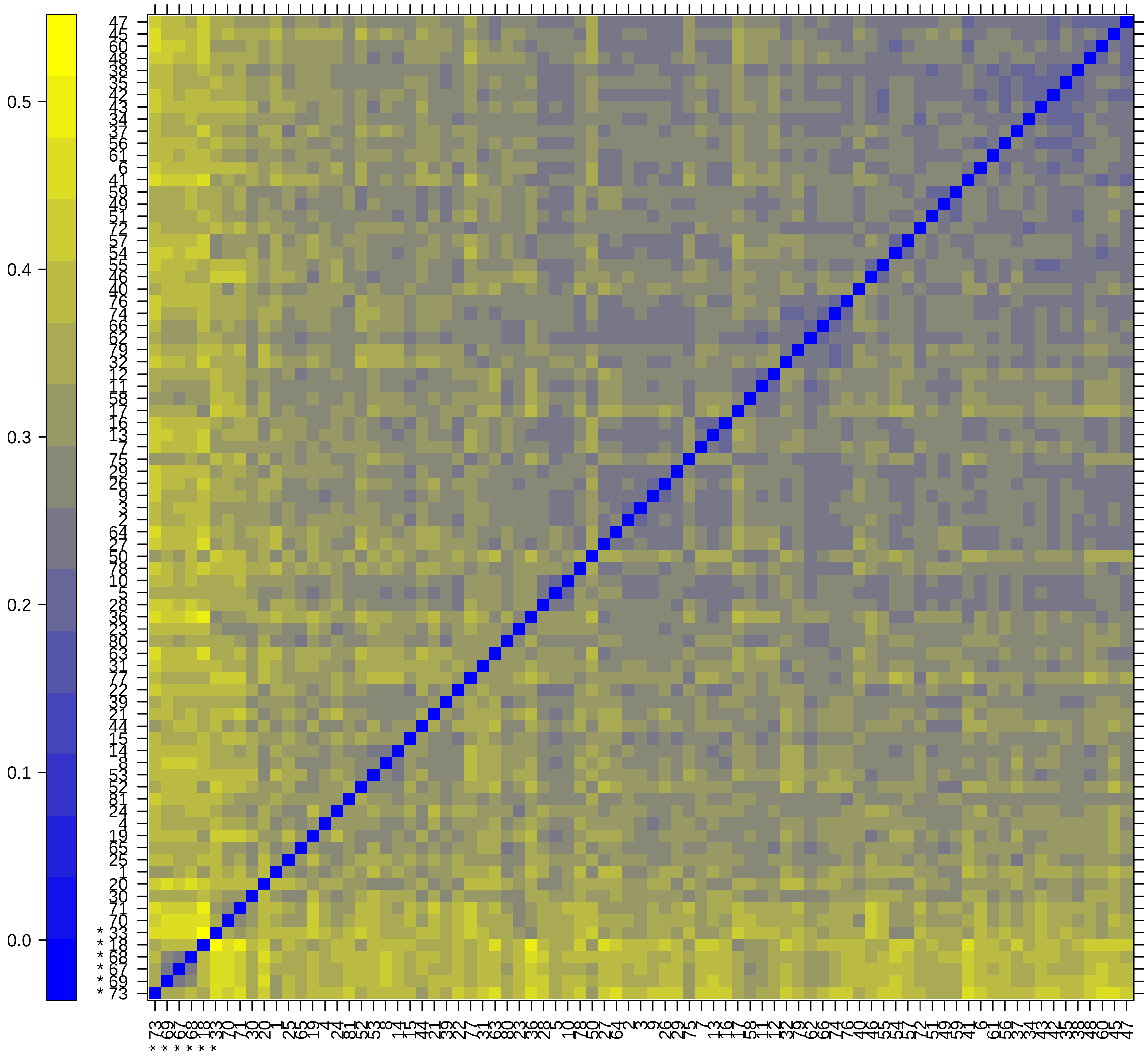
0.5
0.4
0.3
0.2
0.1
0.0
47 45 60 48 38 35 42 43 34 37 56 61 6 41 59 49 51 72 57 54 55 46 40 76 74 66 62 79 32 12 11 58 17 16 13 7 75 29 26 9 3 2 64 27 50 78 10 5 28 36 23 80 63 31 77 22 39 21 44 15 14 8 53 52 81 24 4 19 65 25 1 20 30 71 70 33 18 68 67 69 73
